# NMR metabolomics as a complementary tool to brix-acid tests for navel orange quality control of long-term cold storage

**DOI:** 10.1038/s41598-024-77871-z

**Published:** 2024-12-03

**Authors:** Keeton H. Montgomery, Aya Elhabashy, Maria Del Carmen Reynoso Rivas, Gurreet Brar, V. V. Krishnan

**Affiliations:** 1 Department of Chemistry and Biochemistry, California State University Fresno, Fresno, CA 93740 USA; 2 Department of Plant Science, California State University Fresno, Fresno, CA 93740 USA; 3 Department of Pathology and Molecular Medicine, University of California Davis School of Medicine, Sacramento, CA 95817 USA

**Keywords:** Nuclear magnetic resonance (NMR), Metabolomics, Brix/Acid tests, Cold-storage, Citrus, Metabolomics, Analytical chemistry

## Abstract

Quality control plays a crucial role in maintaining the reputation of agricultural organizations by ensuring that their products meet the expected standards and preventing any loss during the packaging process. A significant responsibility of quality control is conducting periodic product assessments. However, subjective interpretation during physical inspections of fruits can lead to variability in reporting. To counter this, assessing total soluble solids (Brix) and percent acidity (Acid) can provide a more objective approach. Nevertheless, it is essential to note that many fruit metabolites can impact these parameters. Nuclear magnetic resonance (NMR) spectroscopy, particularly ^1^H-NMR, has become a popular tool for quality control in recent years due to its precision, sample preservation, and high throughput analysis. This manuscript investigates if the standard Brix/Acid tests are directly related to the levels of metabolites during cold storage. Using citrus as the model system, a metabolomics analysis was conducted to identify patterns in the cold storage metabolite profiles of the juice, albedo, and flavedo tissues. The results show that Brix (or total dissolved solids) correlates well with sucrose, glucose, and fructose levels and moderately with choline levels. Acid (percent acidity) levels displayed a negative correlation with both fructose and choline levels. Interestingly, the formate levels were susceptible to storage time and directly related to Acid measurements. This study suggests metabolomics could be a complementary technique to quality control of fruits in cold storage, especially with cost-effective desktop NMR spectrometers.

## Introduction

Citrus fruit is a major agricultural contributor, producing over 47 million tons of fresh fruit in 2022, with the United States producing 3.6 million tons^1^. Citrus farmers and production plants lose yield yearly due to pre- and postharvest conditions such as citrus greening disease, insect damage, infestation by postharvest pathogens, and physiological disorders that manifest on the fruit during long-term cold storage^2–4^. Pack houses must implement quality control practices to prevent sending fruits with these defects to the market^5^. Ideally, fruits are processed, packaged, and shipped to the market as soon as they arrive at the pack-house after harvest. Due to factors such as processing line thresholds, storage availability, logistical thresholds, and market conditions, fruit can remain in cold storage for an indefinite time before it reaches the market^6,7^. For this reason, packhouses carefully monitor the quality of products in cold storage to ensure the market receives products of optimal quality. This ensures that pack-houses maintain their reputation with consumers, maximizing profit margins for future harvest seasons.

Traditional, non-invasive quality control methods include a physical inspection of the fruit and the quantification of decay. Still, many of these methods are subjective and will vary between individual inspectors^3^. A more objective and repeatable approach to quality control is the assessment of Brix, which accounts for the total soluble solids (TSS) in the juice and the percent of Acid in the juice^8^.

These assessments are commonly used in the field to determine the optimal window for harvest, but they can also be used in the pack-house to assess quality for long-term storage of fruits^3,8^. Brix, or TSS, in fruit accounts for all metabolites soluble in aqueous media, including glucose, sucrose, fructose, myo-inositol, amino acids, organic acids, nucleotides, and many other water-soluble metabolites such as trigonelline, synephrine, and choline^8,9^. Brix is commonly measured by determining the specific gravity of citrus juice or by measuring the refractive index of the juice using a portable refractometer^3^. As citrus fruit matures on the tree, carbohydrate levels in the juice are expected to increase, increasing Brix. Measuring the percentage of acids in citrus juice is typically achieved via sodium hydroxide titration using phenolphthalein as an indicator^8^. The citrus industry reports the titration results as the percentage of citric acid, the most abundant acid in citrus juice, but other organic acids in the juice, such as ascorbic acid, succinic acid, malic acid, formic acid, and acidic amino acids will influence the titration results. As citrus fruit matures on the tree, citric acid levels typically decrease, and reported levels of percentage acid should also be reduced^8^. These general trends are expected as fruit matures on the tree. However, what happens to TSS and Acid levels after the fruit is harvested depends on factors such as storage conditions and commodity type^10^.

Since Brix and Acid measurements are essentially a total measurement of all metabolites within their respective category, assessing these parameters could lead to ambiguity. Citrus commodities can contain distinct metabolites that serve as specific maturity indices while the fruit is stored. For instance, Kim et al. evaluated Satsuma mandarins at five different maturity phases for three and a half months and found differential trends for various amino acid levels^11^. However, in this study, the fruits remained on the tree and were monitored immediately after harvest throughout their respective season. Sun et al. stored Hirado-Buntan Pummelo fruit for 132 days at ambient conditions, assessing carbohydrates, organic acids, and amino acids during storage. A general downward trend of citric acid, malic acid, and carbohydrates was observed during storage^12^. However, not all citrus commodities will have the same storage parameters, with most requiring cold storage conditions^13^. Tang et al., stored Powell oranges for 60 days at ambient and cold storage conditions. However, there was little change in the metabolite composition of the cold-storage fruit^14^. Several other metabolomics temporal studies have been performed on citrus fruit, but the objectives of these studies were to determine metabolic changes induced by commercially practiced treatments^15–17^.

As most citrus metabolomics studies aimed to gain insight into physiological changes in the fruit under various conditions, several studies have attempted to use metabolomics for citrus quality control assessment^18–20^. In particular, ^1^H Nuclear Magnetic Resonance (NMR) spectroscopy has gained attention as a tool to monitor food quality in recent years^21^. NMR spectroscopy is a high throughput, non-destructive, and precise analytical technique, making it a viable tool for quality control assessment^22^. It has been demonstrated that NMR spectroscopy can be used as a quality control tool for other food and beverage products besides citrus, such as wine, extra virgin olive oil, coffee, and beer^23–27^.

Navel oranges are seeded, sweet oranges that are easy to juice and have a growing popularity^28,29^. In this work, we utilized ^1^H NMR spectroscopy to identify specific metabolomic trends associated with the maturity of Navel oranges in cold storage. In particular, the focus is to determine metabolites that correlate with the Brix or Acid measurements and investigate complementary ^1^H NMR-based metabolomics measurements. The rind of the fruit (flavedo and albedo tissues) and the juice were analyzed via NMR spectroscopy. At the same time, the assessment of Brix and Acid was also performed on the fluid for comparison. Our results indicate that time-dependent variation of formate negatively correlates with Brix (correlation coefficient -0.91) while correlates positively with total percentage of Acid (correlation coefficient 0.79). We also identified that in the case of the juice sample, the time-dependent variations of Brix show expected positive correlations with fructose (correlation coefficient 0.89), glucose (0.87), and sucrose (0.83), with additional moderate correlations with choline (0.76), trigonelline (0.75) and myo-inositol (0.77). These results broadly indicate the feasibility and the potential of metabolite profiles that could be used as an alternate approach to investigate fruit quality.

## Results/discussion

### Time-dependent metabolomics changes due to cold storage

**Fig. 1:**
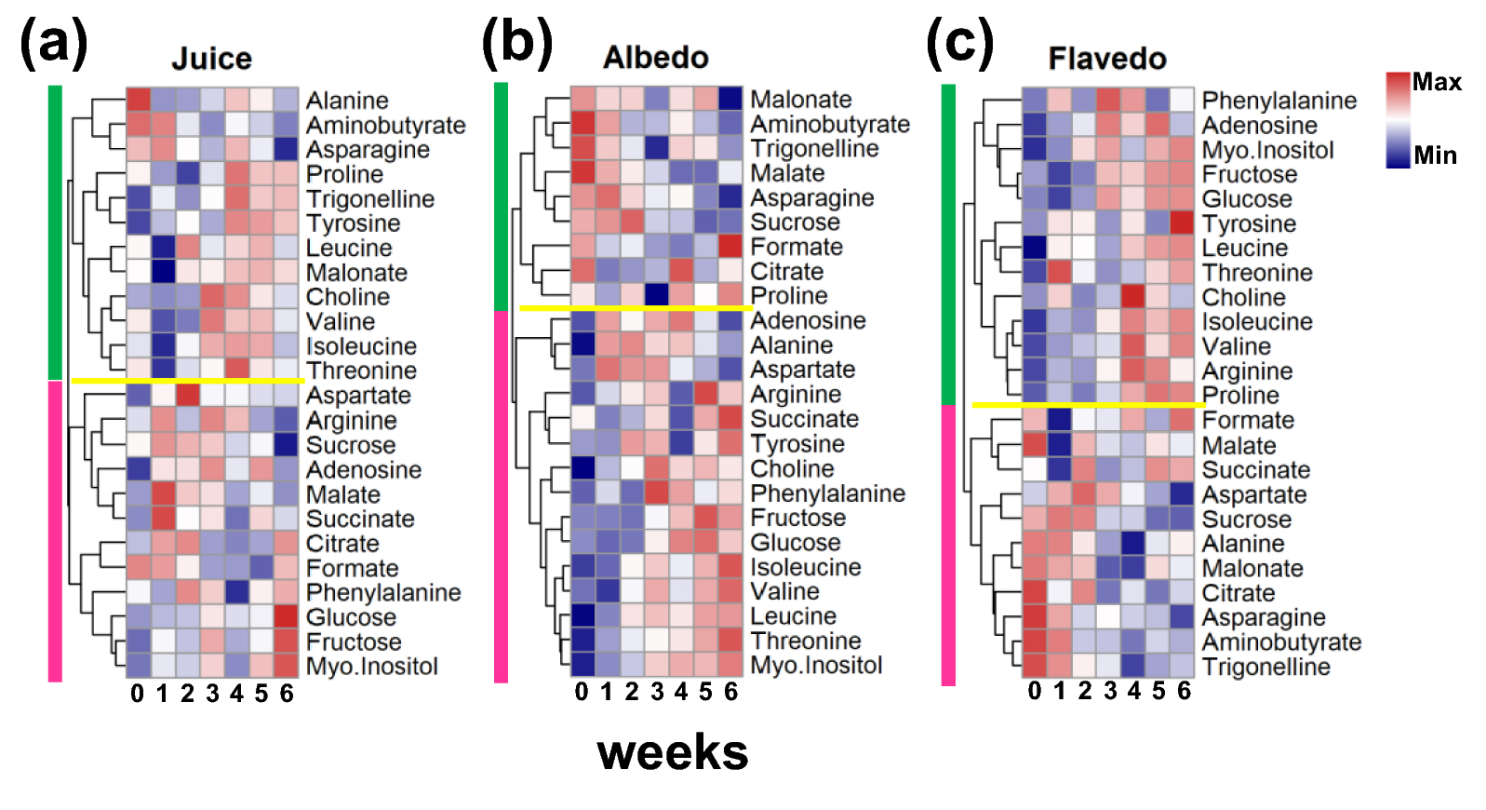
Week-dependent distribution of detected metabolite concentrations. Hierarchical clustering of the citrus metabolites shown as a heatmap for the (**a**) juice, (**b**) albedo tissues, and (**c**) flavedo tissues. Metabolite concentrates are clustered on a Euclidean distance metric, and the final concentrations (mM) are scaled for visual presentation. The two major clusters in each plot are shown as vertical bars on the left.

Twenty-four metabolites were identified, entailing four carbohydrates, eleven amino acids, five organic acids, and four other molecules. Figure 1 shows the overall time-dependent variations in the concentrations of the metabolites for the juice (Fig. 1a), albedo tissue (Fig. 1b), and flavedo tissue (Fig. 1c). Tables S1, S2, and S3 contain metabolite concentration data collected for the juice, flavedo, and albedo samples. The clustering of metabolites does not generate a notable trend in a collection of grouped metabolites. Figure S1 shows the representative examples of the ^1^H NMR spectra of juice (S1a), albedo (S1b), and flavedo (S1c) samples, along with the peak identification of the metabolites. The experimental design includes five replicate measurements in each condition to ensure reproducibility. A correlation analysis of the replicates indicates a correlation coefficient > 0.97 (representative analysis of juice, albedo, and flavedo samples, supporting information Fig. S2). Principal Component Analysis (PCA) plots generated from the time-dependent (weeks) metabolite data of the juice, flavedo, and albedo can be depicted in Figs. S3a, S3b, and S3c. For every tissue, cluster overlap exists between the controls and the first three weeks of data. Still, the clusters for weeks 4, 5, and 6 are distinct from those of the controls, indicating a shift in the metabolite composition during the fourth week of storage. The hierarchical clustering (Fig. 1) typically shows two major metabolite clusters (vertical bars) in juice, albedo, and flavedo samples. However, the metabolites within these clusters are not the same across the different samples.

**Fig. 2:**
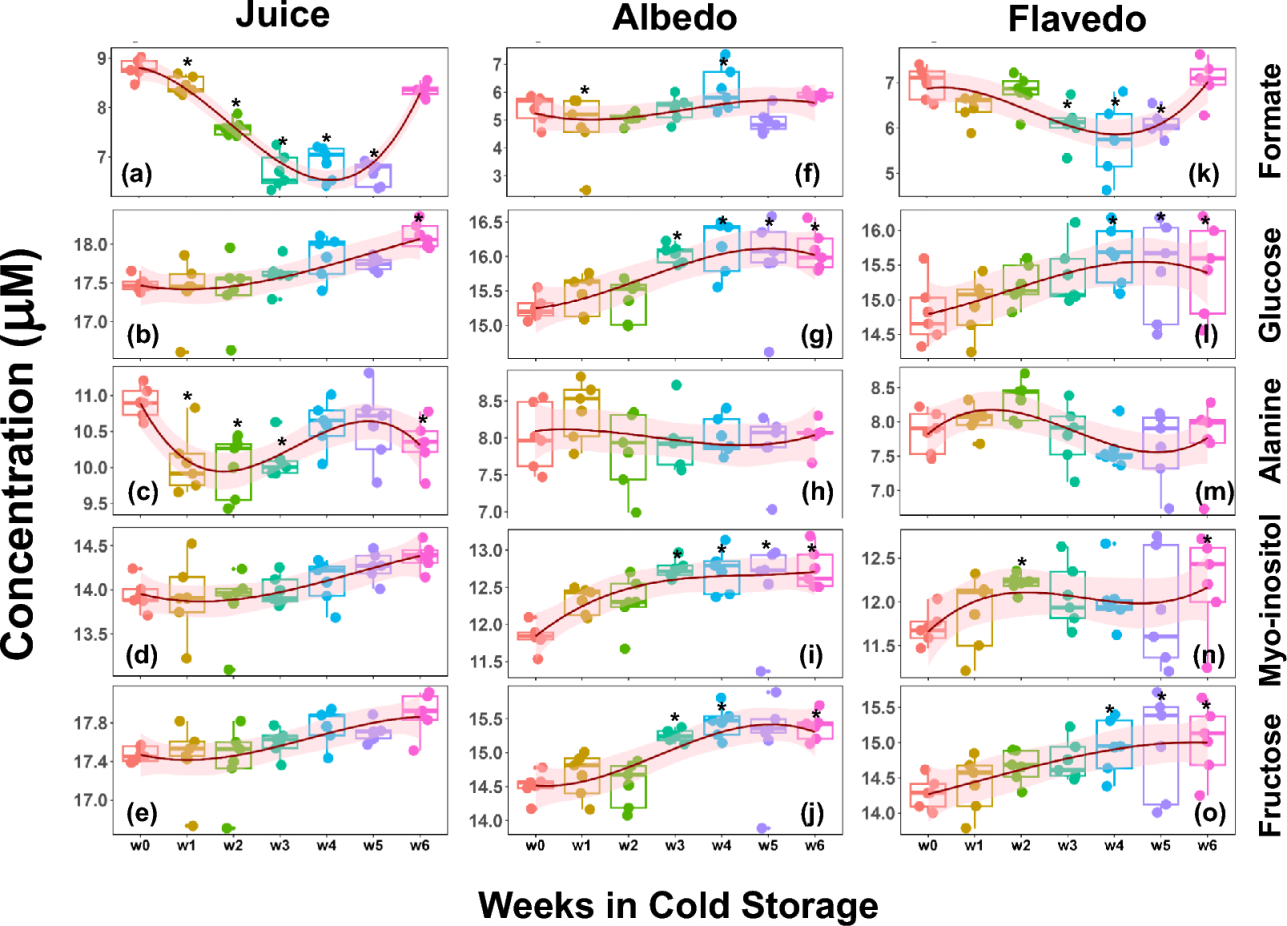
Metabolomic profiles due to cold storage. Metabolites that show significant alteration in the cold storage in at least one of the samples (juice or tissues albedo/flavedo) or during the weeks in cold storage. The box-whisker plots for the metabolites formate, glucose, alanine, myo-inositol, and fructose (rows) are plotted in juice, albedo, and flavedo (columns). A spline fit as a function of weeks is shown to visualize a trend. Metabolites that show significant changes (|fold-change|> 1.5 and p.value < 0.05) with respect to the week zero (w0) are marked with ‘*.’

The discriminate analysis does not show a group of metabolites having a specific trend with increasing time at the cold storage (Figs. 1 and S3). As a next step, particular metabolites that are differentially altered due to cold storage with reference to the 0^th^ week (as control) are evaluated using a multivariate approach. For each of the sample conditions (juice, albedo, or flavedo), a metabolite is considered altered significantly if the |fold-change| (> 1.5) and FDR-adjusted p-value (< 0.05). Due to cold storage, five metabolites (formate, glucose, alanine, myo-inositol, and fructose) are differentially altered in juice or tissue samples (Fig. 2). Formate tends to be a metabolite showing significant variations in juice and flavedo samples (*vide infra*). The variation in the levels of alanine is notable in the cold storage of the juice (Fig. 2c). Myo-inositol and fructose show significant differences with the number of weeks in the cold storage but do not show many alterations in the juice samples.

### Differential alterations of metabolites due to cold storage.

#### Juice samples

**Fig. 3:**
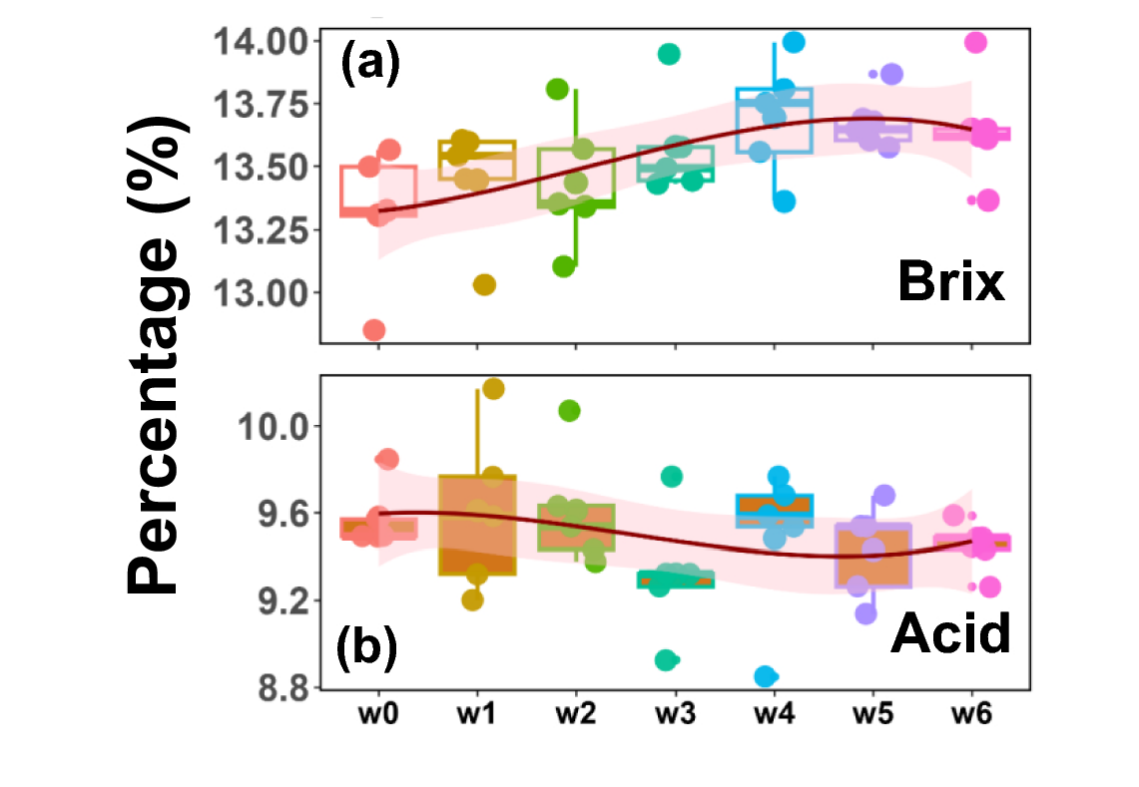
Weekly changes in the Brix and Acid measurements in cold storage. Variation in the Brix (**a**) and Acid (**b**) measurements as a function of the number of weeks in the cold. The box-whisker plots and a spline fit as a function of weeks are shown to visualize a trend.

Figure 3 and Table S4 present the weekly Brix and percent Acid data. As expected for citrus fruit maturing on the tree, the stored fruit showed an increasing trend for Brix levels and a decreasing trend for percent Acids. However, based on the statistical analysis conducted with respect to the start of the experiments (w0), the fold changes in either Brix or Acid are insignificant. Organic acids, mainly citric and malic acids, dominate the contributions to the Acid measurements^30^. As sensitive HPLC measurements are needed to differentiate the various organic acids in the fruit sample, no additional measurements were performed to distinguish the different organic acids^31^. The concentrations of glucose, fructose, and myo-inositol (Fig. 2) did increase during storage, which supports the analysis of Brix (Fig. 3). However, the percent Acid values reported are based upon percent citric acid calculations determined from titration results, similar to the industrial protocol. Given the juice metabolite data, there was a minor change in weekly citrate concentrations, with a slightly increasing trend. Considering that titration results are governed by the initial sample pH, pKa values of organic acids and bases in solution, and the concentration of these species, reporting percent Acids as percent citric acid can lead to ambiguity.

The most notable trend observed in the juice was formate (Fig. 2a). Formate levels dropped drastically during the first three weeks, and concentrations remained constant at around 0.1 mM for the two weeks after. During the sixth week of storage, the levels of formate tripled, which was entirely unexpected. To our knowledge, the role of formate in citrus has not been thoroughly explored. However, the part of formate in higher plants includes the synthesis of purines and several amino acids^32,33^. The weekly data of adenosine, a purine, for the juice, flavedo, and albedo samples are shown in Fig. S4. The trend in adenosine concentrations increased weekly, but there was a drop in concentration during the sixth week of storage. Although the variability for weekly adenosine concentrations was much higher than that of formate, this general trend may explain the observed formate levels. However, the origin of formate during the sixth week of storage is unclear. To our knowledge, the synthesis of formate in citrus fruit has not been thoroughly explored. Still, it has been proposed to be a glycolysis product in potato tubers, similar to many bacteria species^34,35^. Although glucose levels did not drop during the fifth week of storage, there was a decrease in sucrose concentrations, which supports this observation (Fig. 2b, g and l; and Tables S1–S3).

#### Tissue samples (albedo and flavedo)

**Fig. 4:**
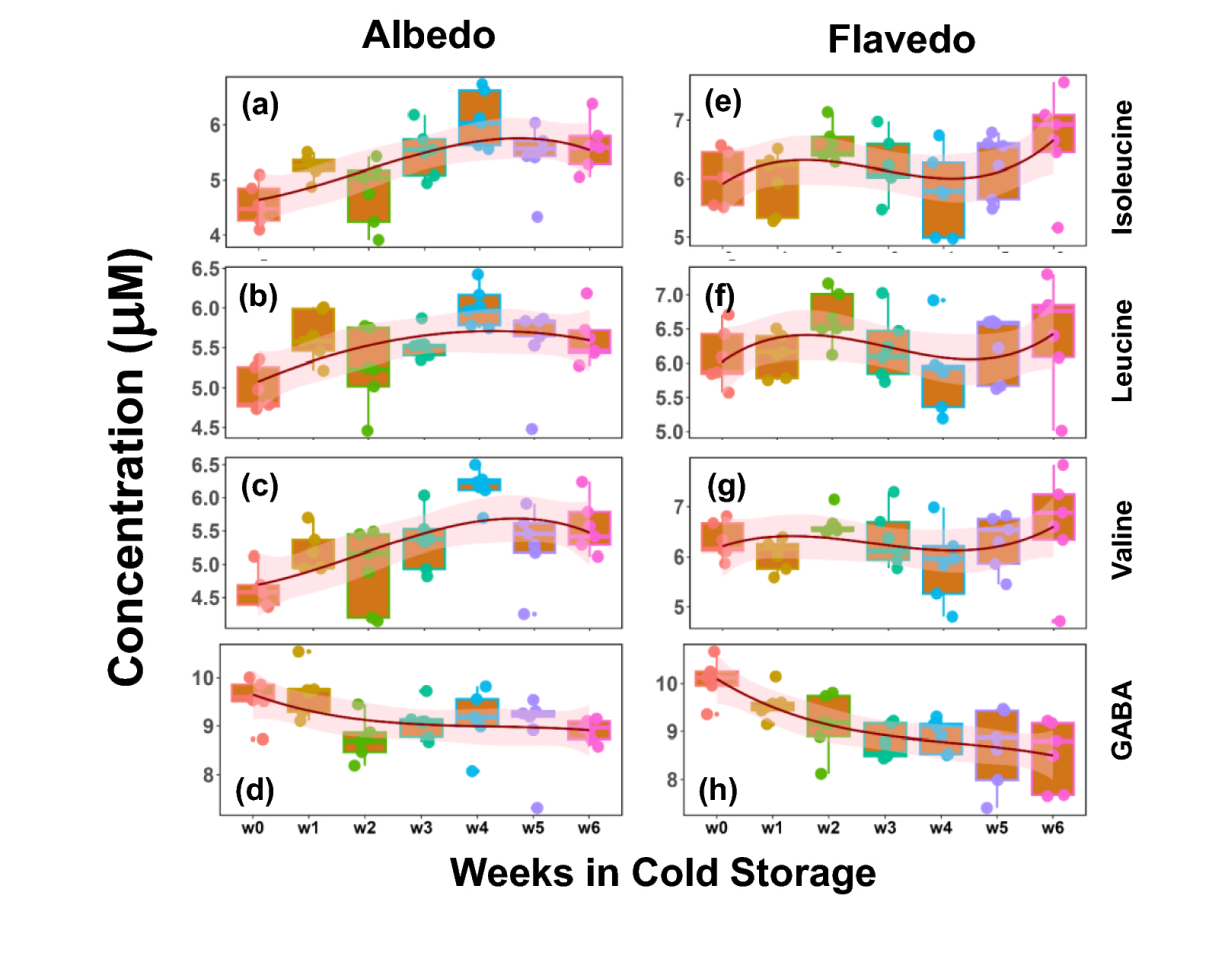
Metabolomic profiles due to cold storage for albedo and flavedo tissues. Time-dependent trends of metabolites in albedo and flavedo during the weeks in cold storage. The box-whisker plots for the metabolites isoleucine, leucine, valine, and GABA (rows) are plotted for albedo and flavedo tissues (columns). A spline fit as a function of weeks is shown to visualize a trend.

Several notable trends were observed in the albedo and flavedo tissues; glucose, fructose, and myo-inositol concentrations increased weekly for the albedo tissue. In contrast, the sucrose levels decreased, similar to the findings of Tang’s assessment of Powell oranges (Fig. 2)^14^. These observations imply that sucrose was hydrolyzed during storage. Concentrations of isoleucine, leucine, valine, and all branch-chained non-polar amino acids also had a weekly upward trend in the albedo (Fig. 4a–c) and flavedo tissues (Fig. 4e–g). These amino acids have been demonstrated to have many biological functions in plant tissues, such as providing drought resistance, accumulation during abiotic stress, and supporting plant growth^36–38^. As the fruit remains in cold storage, these amino acids may accumulate in response to the inevitable water loss. However, all three amino acids accumulated the highest concentration during the fourth week of storage, followed by a drop in concentration during the fifth week.

Several metabolic trends in the flavedo tissue were noticed as well. The levels of glucose, fructose, and myo-inositol increased. In contrast, the levels of sucrose decreased, just as was observed in the albedo tissue (Fig. 2). The metabolite 4-aminobutyrate (GABA) levels decreased during the first week of storage in both albedo (Fig. 4d) and flavedo tissues (Fig. 4h), as the accumulation of GABA is usually associated with plant stress^39^. Cercós and Lourkisti reported decreased levels of GABA on mature mandarins ripening on trees compared to fruit harvested earlier in the season; however, the fruit was analyzed immediately after harvest and was not kept in cold storage^40,41^. Cercós also reported constant levels of glutamate, suggesting the activation of the GABA shunt during acid catabolism^40^. Although our analysis did not detect glutamate, citrate levels decreased during the first week of storage (Tables S1–S4), indicating the activation of the GABA shunt and an explanation for the observed levels of GABA. Aspartate levels in the flavedo also had an interesting trend (Tables S1–S4). Isoleucine, which can protect against dehydration, can be produced in the aspartate pathway^37,42^. Although observed levels of isoleucine in the flavedo did not correlate with aspartate levels, isoleucine in the albedo tissue did have a weekly upward trend.

#### Correlation of Brix-Acid tests and metabolite time dependence.

**Fig. 5:**
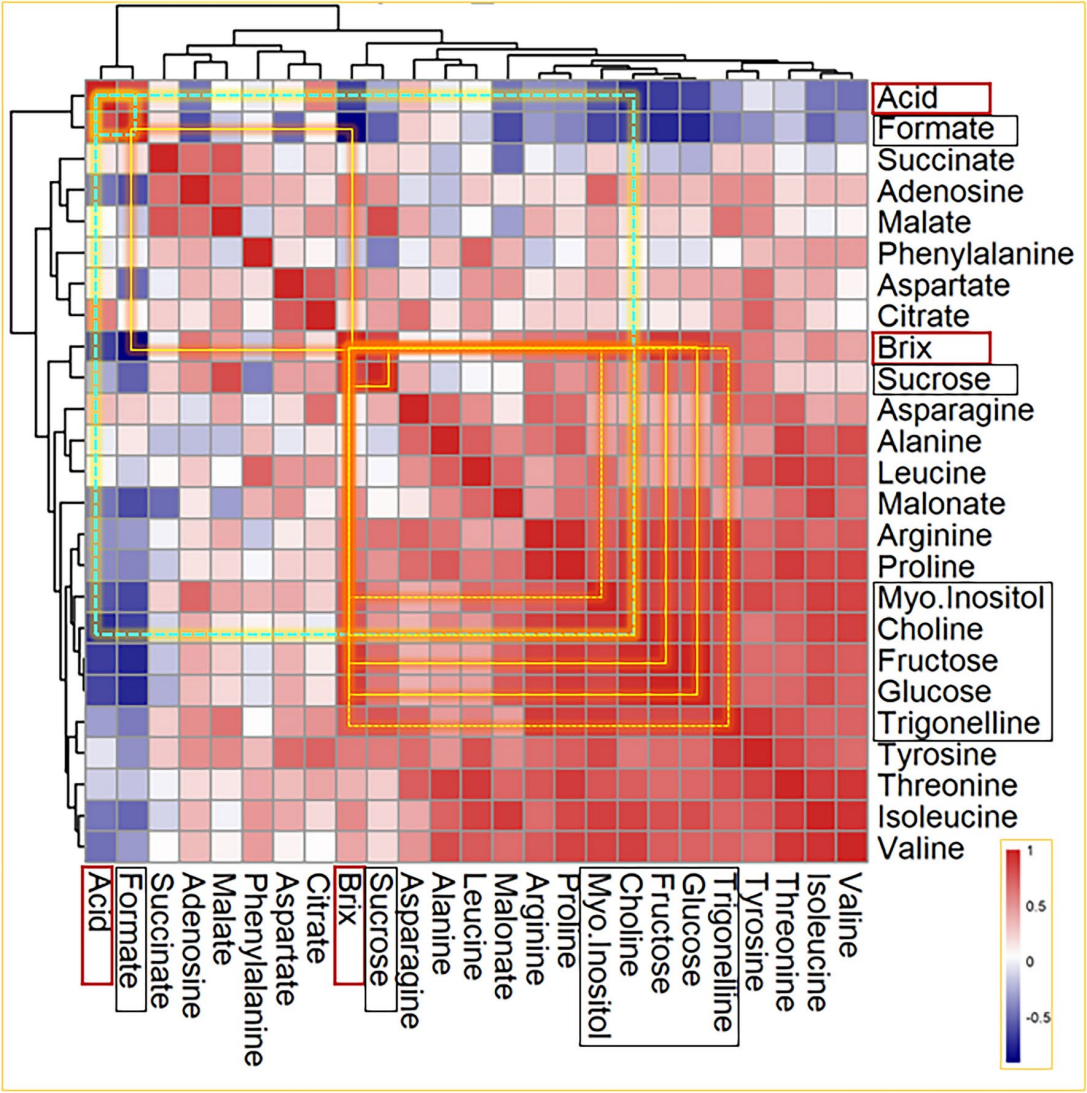
Correlation between Brix/Acid and metabolites: Heatmap depicts the correlation (Pearson) of the time-dependent variation of the Brix and Acid tests and the NMR-measured metabolites. The dendrograms (hierarchical clustering by Euclidean distance) identify sub-groups of correlated measurements. Metabolites that have a positive or negative correlation with Brix are shown as yellow squares, and with Acid are shown as green squares. Continuous lines have correlation coefficients > 0.8 and p-values < 0.05, while the dashed lines 0.7 < correlation coefficient < 0.8 and 0.05 < p-values < 0.1.

Though Brix and Acid tests are considered standard in the postharvest industry, a correlation analysis is performed on how each metabolite’s time-dependent cold storage profile relates to the Brix and Acid profile. This approach provides a means to quantify the similarity between the time-dependent trends of the various metabolites. In particular, the focus of the analysis is to identify metabolites that have a profile similar to the profiles of Brix or Acid tests. Figure 5 shows the correlation plot of the various metabolites, including Brix/Acid measurements. A correlation matrix for the albedo and flavedo is shown in the supporting material (Fig. S5). The correlation matrix was further subjected to hierarchical clustering to identify the ground of metabolites with time-dependent cold storage trends. The correlation coefficients were estimated using the Pearson metric, and the hierarchical clustering of the coefficients was performed with an Euclidean distance.

As expected, Brix measurement has a positive association with the sugars with a high correlation coefficient and low p-value: fructose (correlation coefficient: 0.89, p-value: 0.01), glucose (0.87, 0.02), and sucrose (0.83, 0.04). These correlations are shown as yellow squares in Fig. 5. Brix measurements also correlate with a moderate correlation with myo-inositol (0.77, 0.07), trigonelline (0.75, 0.08), and choline (0.76, 0.08); shown as dotted squares (Fig. 5). Brix measurements show a negative correlation of − 0.91 (p-value 0.01) with the formate variation with time in the cold storage. The Acid measurements (shown as green squares of Fig. 5) show positive correlations with formate (0.79, 0.06) and a negative correlation with choline (− 0.72, 0.10). Interestingly, formate shows a positive correlation with Acid (0.79, 0.06) and a negative correlation with Brix (− 0.91, 0.01). On the other hand, choline, which shows a negative correlation with Acid (0.72, 0.10), offers a positive correlation with Brix (0.76, 0.08). The role of formate was discussed previously (Fig. 2). Though the correlation of choline, a water-soluble vitamin, is moderate, it emerges as a potential metabolite as it directly correlates with Brix and inversely with Acid—a trend opposite to formate.

## Conclusion

Postharvest chilling injury is a common issue affecting many citrus species. It occurs when fruits are exposed to low temperatures during transportation and storage, leading to physiological and biochemical changes^43–49^. This can result in symptoms such as pitting, decay, and off-flavors, impacting the quality and shelf life of the fruit. Despite being a challenge for the industry, efforts are continually made to develop strategies to minimize chilling injury and ensure that consumers receive high-quality citrus fruits.

Refrigeration is commonly used to prolong the shelf life of fruits^50^, but it can also cause chilling injury in certain fruits and vegetables^10^. Citrus fruits are known for tolerating these conditions, making them good candidates for storage^51^. Although techniques like hot water or molybdenum dips have been proposed to reduce postharvest chilling injury, they may also adversely affect the quality and shelf life of fruits and vegetables^52^. This study demonstrates the effectiveness of the metabolomics technique using cold storage of citrus as an example.

To investigate biomarkers that may serve as complementary to industrial standards of Brix and Acid measurements, this study identifies several metabolites that may serve the purpose. In particular, formate emerges as a sensitive metabolite that could be used as an indicator for cold storage quality marker of citrus. In addition to showing significant alterations in its levels, a correlation analysis suggests that the levels are inversely related to Brix measurements and connect directly to the Acid measurements. A similar differential trend for choline (directly associated with Brix but inversely to Acid) is also noted. NMR spectroscopy has not been widely used for postharvest examinations of fruits, especially citrus fruits. However, this study shows the potential for NMR techniques to be considered as a supplementary tool in these investigations.

Several metabolic trends were observed for each tissue, namely the flavedo and albedo tissues. However, the reader should be advised not to assume the same trends will be monitored for other citrus varieties under the same experimental conditions^39^, and additional experiments must be warranted. Even fruit of the same array harvested from different geographic regions can be expected to have distinct metabolic profiles^53^. Other factors that can cause variability in metabolic profiles include harvest year, rootstock, and underlying abiotic stresses^13,53,54^. Further experiments involving genomic or proteomic studies are needed to understand the biochemical pathways responsible for the observed changes in the metabolite levels. Nonetheless, we have demonstrated that NMR Spectroscopy can be utilized as a tool to identify specific metabolic trends of Navel fruit in cold storage.

## Material and methods

### Chemicals and reagents

All chemicals, reagents, and supplies were purchased from Fisher Scientific.

### Plant materials

Navel Fruit of comparable size and color were harvested in May of 2022 from Fresno State Orchards (with permission as one of the authors in the plant sciences department that maintains the orchard). The fruits were stored at 5 °C for 6 weeks after harvest. Five fruit replicates were processed for analysis on the initial harvest date and once a week for six weeks after harvest. Once the fruits are removed from the storage, all the samples are prepared for the analysis within 12 hours.

### Sample preparation

Sample preparation was adapted from Slisz and Kim with some modifications^9,55^.

#### Juice samples

50 mL of juice from each replicate was collected, and the pulp was removed using a kitchen strainer. 4 mL of the strained juice was transferred to 3 kDa molecular weight cutoff filters; then, the samples were centrifuged for 30 min at 18,000 g and 20 °C. 1 mL of the filtrate was transferred to 1.5 mL Eppendorf tubes with a 1 mm hole drilled on the cap for solvent sublimation during lyophilization. The samples were then frozen and lyophilized for 48 h. Samples were suspended in 700 μL of D_2_O buffer solution containing 90 mM KH_2_PO_4_, 0.2 mM imidazole (IMZ), and 0.05 mM sodium 3-trimethylsilyl [2,2,3,3, d4] propionate (TSP) with the pH adjusted to 6.8. Samples were vortexed for 20 s and then centrifuged for 30 min at 18,000 g. 600 μL of the supernatant was then transferred to 5 mm NMR tubes.

#### Tissue samples

From each replicate, 1.0 g of both the albedo and flavedo tissues were separated using sterile scalpels. Samples were finely ground with a mortar and pestle with liquid nitrogen. 250 mg of the ground tissue was transferred to 5 mL Eppendorf tubes. 3 mL of reagent-grade methanol was added to the Eppendorf tubes, and the samples were sonicated for 10 min. The samples were centrifuged for 30 min at 18,000 g and 20 °C. The supernatant was transferred to clean 5 mL Eppendorf tubes, and the methanol evaporated for 48 h in a fume hood. The samples were lyophilized for 48 h to remove any remaining solvent. After lyophilization, samples were suspended in 700 uL of D_2_O buffer solution containing 90 mM KH_2_PO_4_, 0.2 mM imidazole (IMZ), and 0.05 mM sodium 3-trimethylsilyl [2,2,3,3, d4] propionate (TSP) with the pH adjusted to 6.8. After vortexing each sample for 20 s, the samples were centrifuged for 30 min at 18,000 g and 20 °C. 600 μL of the supernatant was then transferred to 5 mm NMR tubes.

### Brix and acid assessment

The remaining strained juice was used to determine each sample’s Brix and percent Acid values. The juice density in g/mL was determined by finding the weight of 10 mL of juice using a 10 mL volumetric flask. The density of the juice was divided by the density of water to obtain the specific gravity of the juice. The Brix of the juice was determined by utilizing a linear regression formula generated from the linear relationship between specific gravity and Brix^56^.

The percent Acid levels were determined via acid/base titration using NaOH as the titrant and phenolphthalein as an indicator. 5 mL of the strained juice was transferred to a 25 mL Erlenmeyer flask. Two drops of 0.05% phenolphthalein solution were added to a solution as a titration indicator. The 20 μL portions of 0.1562 N NaOH were slowly added to the solution while vigorously swirling after each portion. Titration was complete when the color change induced by the phenolphthalein persisted after vigorous swirling. The percent Acid was calculated as percent citric acid using the volume of the 0.1562 N NaOH solution required for each titration.

### NMR experiments

For the ^1^H NMR analysis, the extracted samples were resuspended to a final volume of 600 μL in D_2_O, with 0.35 mM sodium trimethylsilyl-2,2,3,3-d4-propionate (TSP) added to each lyophilized, titrated extract for chemical shift calibration. All sample preparations were performed over two days, and samples were subsequently stored at 4 °C. Quantitative ^1^H-NMR spectra were recorded at 600 MHz using a JEOL NMR spectrometer equipped with a liquid N_2_ cooled probe and Z-axis pulsed field gradients at 300 K. One-dimensional ^1^H experiments, with a mild pre-saturation of water resonance, were performed with a 70° pulse angle (Ernst angle)^57,58^. NMR spectra were collected over 1024 transients, with an acquisition time of 2.0 s and a relaxation delay of 3.0 s.

The spectra were processed and analyzed with Chenomx NMR Suite 8.1 software (Chenomx Inc., 2014). Fourier-transformed spectra were multiplied with an exponential weighting function corresponding to a line-broadening of 0.5 Hz. All the spectra were manually phase-corrected baseline optimized, and their chemical shifts were referenced to TSP. Though all the experiments were performed in D_2_O with a mild pre-saturation of the residual H_2_O peak, the spectral region near the water resonance (± 0.1 ppm) was not included in identifying the metabolite peaks. The resulting spectra were analyzed using the PROFILER-Module of Chenomx, and the concentrations of selected metabolites were estimated in all the samples. The combined concentration data was used for the multivariate statistical analysis. The metabolite peaks of the processed spectra were analyzed and assigned to their chemical shifts using the built-in Chenomx. The assigned metabolites were compared and confirmed through chemical shift values of other ^1^H NMR-based metabolomics studies performed in previously published results^27^. The juice, albedo, and flavedo samples’ representative NMR spectra are given in supporting information (Fig. S1).

### Data analysis and metabolite identification

Data was preprocessed using MestReNova by setting the spectrum size to 64 k, performing sin square 90° apodization, manual phasing, baseline correcting, and normalizing and referencing the data with respect to the TSP peak. The spectra were saved as .jdx files for metabolite identification via Chenomx. Preprocessed data was further processed by Chenomx NMR Suite 8.1 by calibrating the TSP and setting the pH for each spectrum. Metabolites were then identified and quantified with the Chenomx NMR Suite 8.1 profiler application.

### Statistical analysis of NMR spectroscopy datasets

Following the experimental design, an established multivariate statistical analysis approach was utilized to identify differentially altered metabolites. The statistical methods are based on established protocols within R-statistical procedures and have been previously applied for metabolomics and other analyses^27,59–61^. A brief description follows.

The replicates at each experimental condition show a correlation of > 0.97 among the samples (Fig. S2). Metabolites differentially altered between groups are determined using linear modeling in the LIMMA package. The control group comprises the albedo, flavedo, and juice of two commodities harvested recently. In contrast, other groups contain the same tissues and juice stored at a cold temperature for ten days, with or without IMZ treatment. Differentially altered metabolites within a single feature are selected and combined with other comparisons to detect differentially altered metabolites using an F-test. Metabolites are considered significant within a given comparison if they pass the threshold of both fold-change (> 1.2) and FDR-adjusted p-value (< 0.05). The R-statistical environment is used for all analyses and plots^62^.

## Supplementary Information

Supplementary Information.

## Data Availability

All data generated or analyzed during this study are included in this published article [and its supplementary information files].
