# Supplementary material for: NMR metabolomics as a complementary tool to brix-acid tests for navel orange quality control of long-term cold storage
Source: Sci Rep. 2024 Dec 3;14:30078. doi: 10.1038/s41598-024-77871-z (PMC11615199; doi:10.1038/s41598-024-77871-z)
Supplement: Supplementary file 1 — Supplementary Information. [file 41598_2024_77871_MOESM1_ESM.pdf]

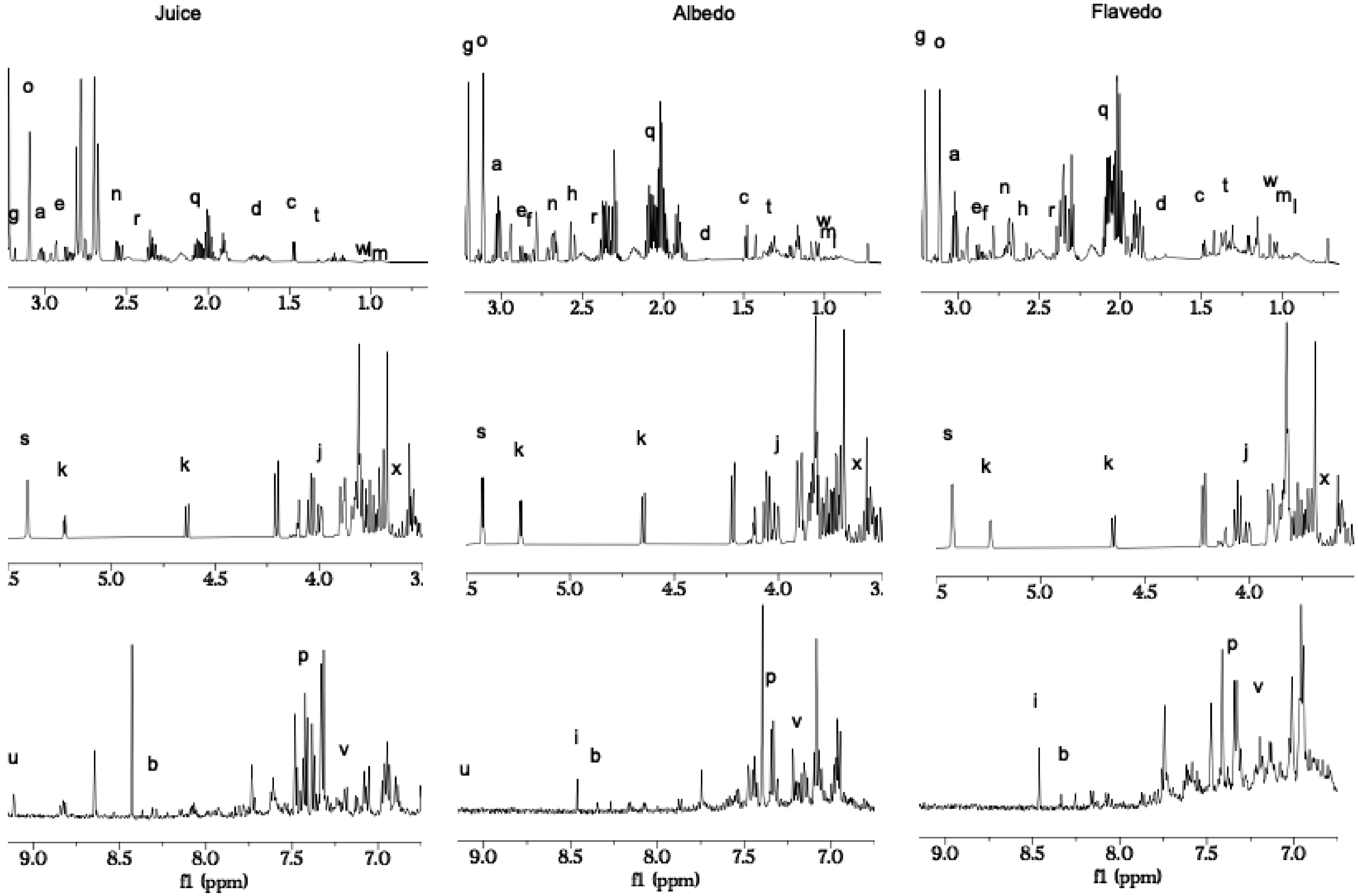

Figure S1: Representative ^{1}H NMR spectra of Juice, Albedo and Flavedo samples. The metabolites are identified as follows. a) 4-Aminobutyrate, b) Adenosine, c) Alanine, d) Arginine, e) Asparagine, f) Aspartate, g) Choline, h) Citrate, i) Formate, j) Fructose, k) Glucose, l) Isoleucine, m) Leucine, n) Malate, o) Malonate, p) Phenylalanine, q) Proline, r) Succinate, s) Sucrose, t) Threonine, u) Trigonelline, v) Tyrosine, w) Valine, x) myo-Inositol

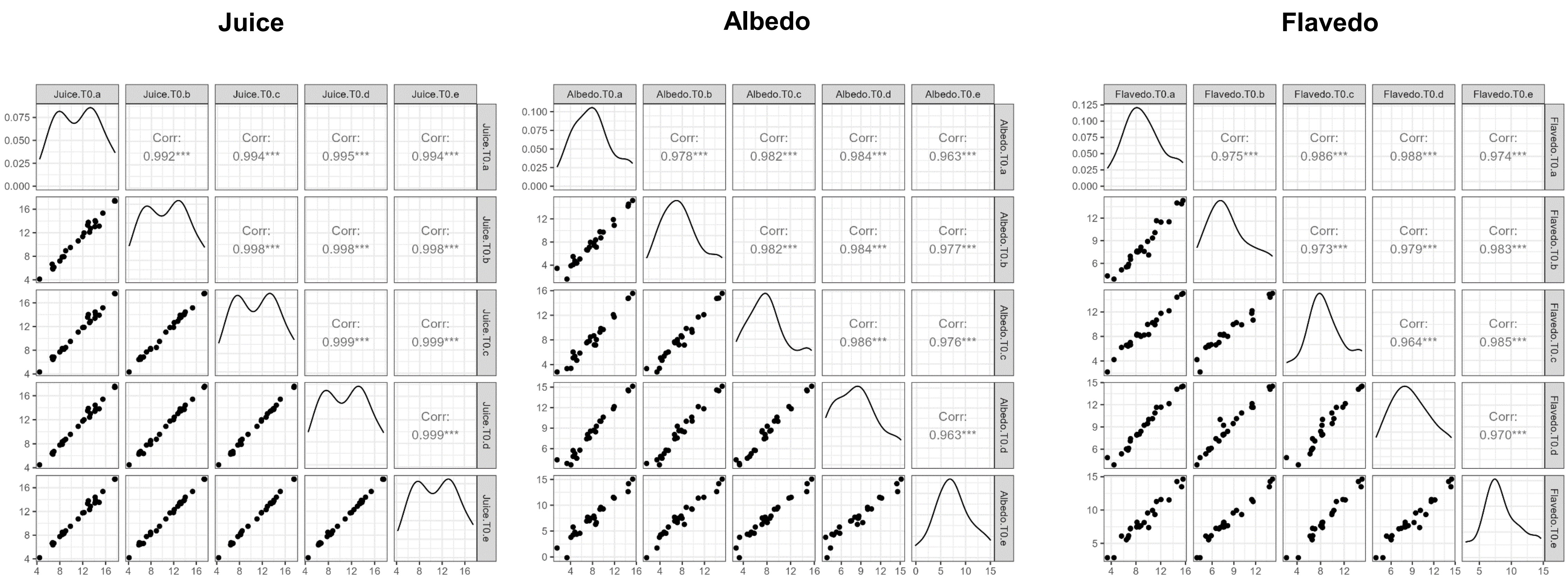

Figure S2: Correlation of the replicate samples. To test the reproducibility of the experimental data, a correlation analysis of the replicate data was performed. Representative replicates of the juice, albedo and flavedo samples (week 0) shows a correlation coefficient of > 0.97.

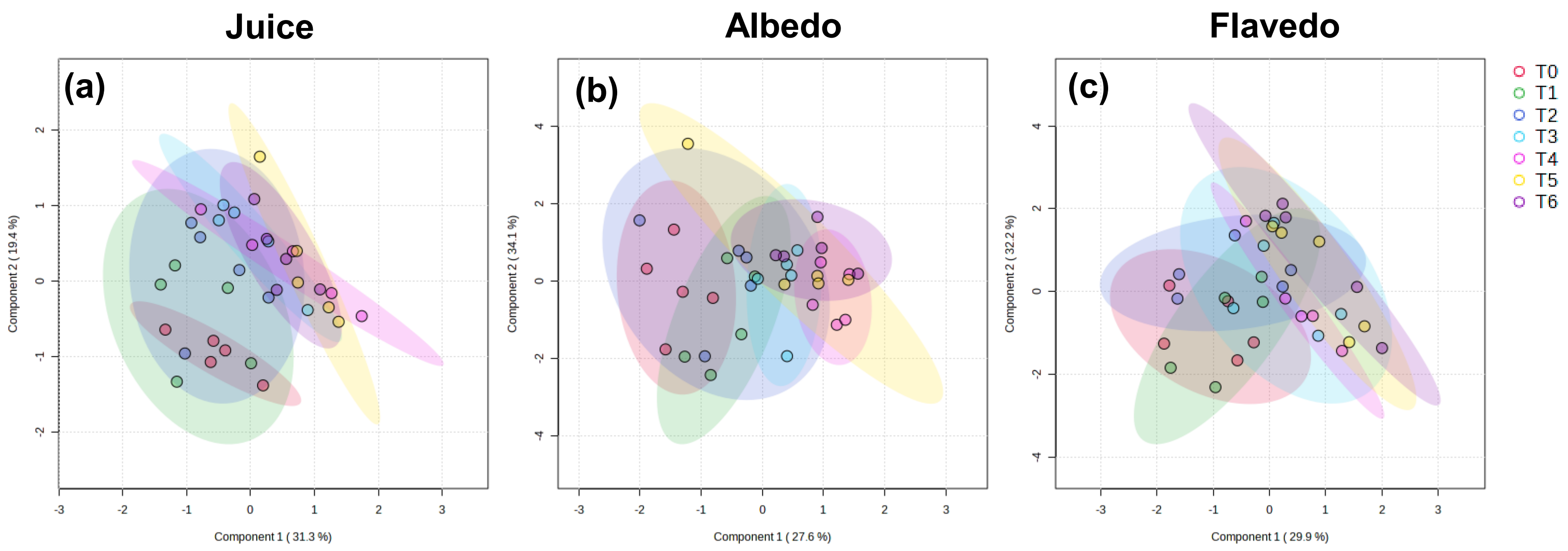

Figure S3: Principal component analysis (PCA). (a) Juice (b) Albedo, and (c) Flavedo. T0 to T6 represents the weeks zero to six.

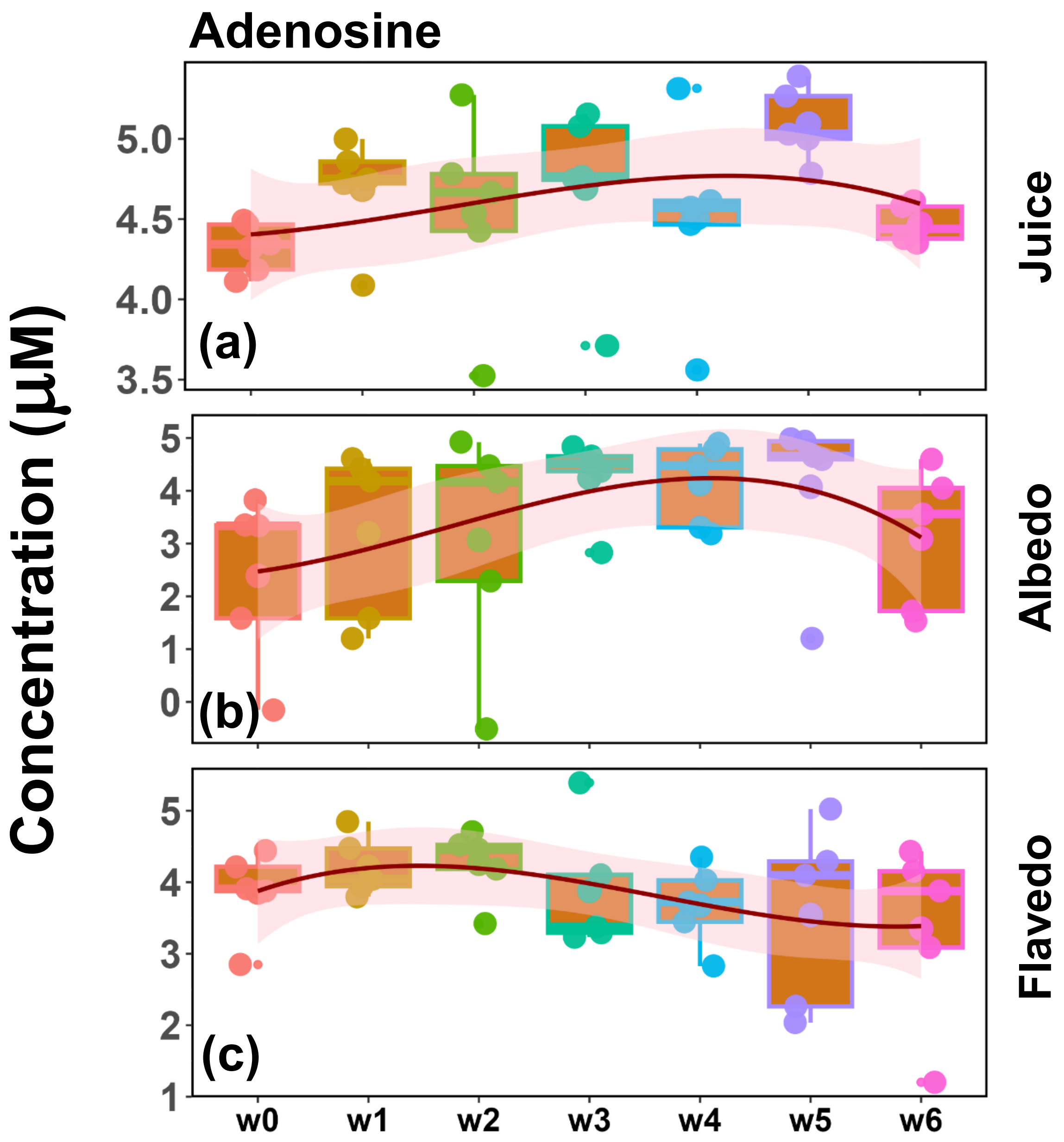

Figure S4: Weekly changes in adenosine levels in juice, albedo, and flavedo.

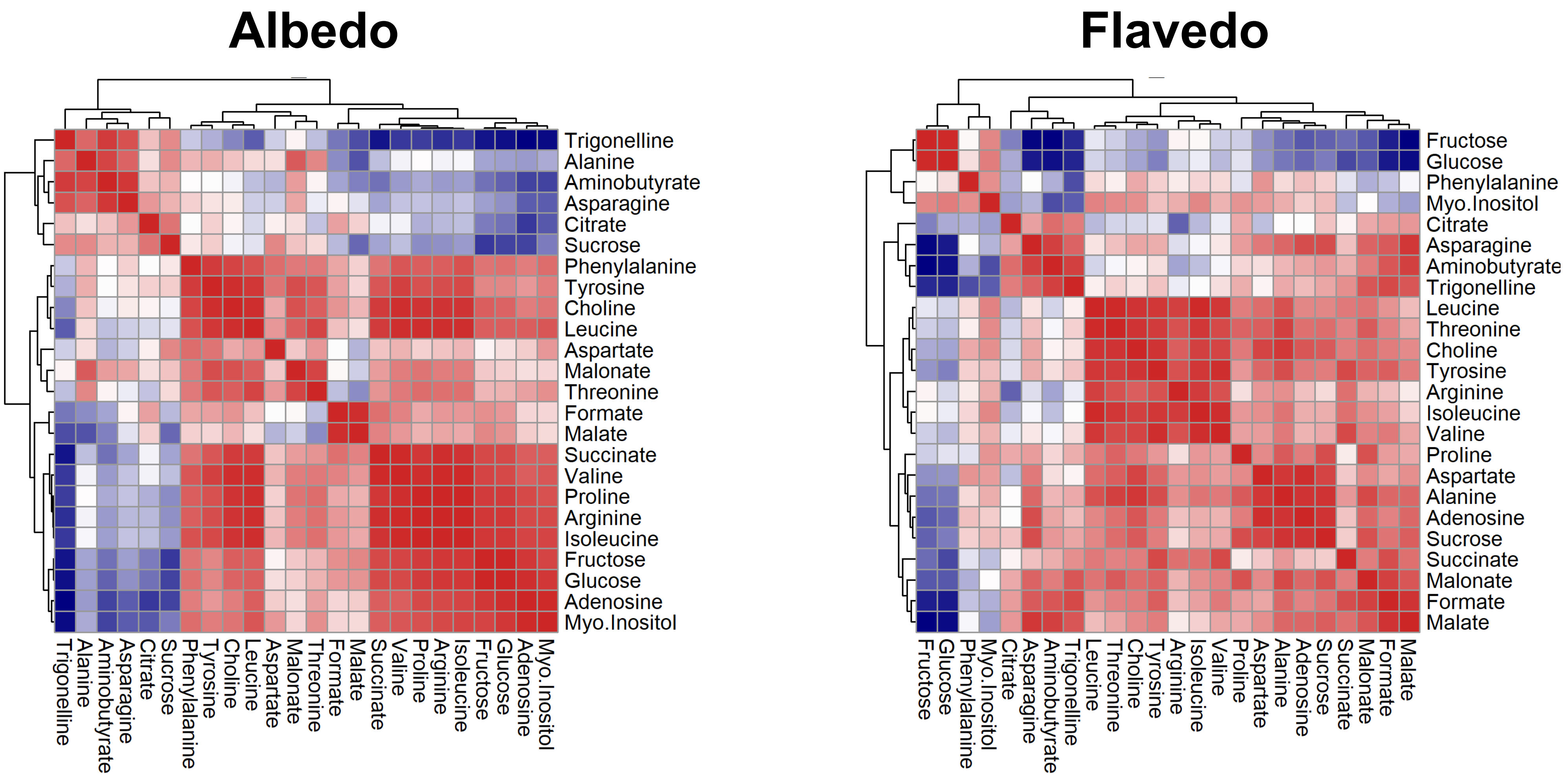

Figure S5: Correlation metabolites: Heat map depicts the correlation (Pearson) of the time-dependent variation of the NMR-measured metabolites.

	Juice						
	W0	W1	W2	W3	W4	W5	W6
**4-Aminobutyrate**	3.45 ± 0.53	3.10 ± 0.79	2.45 ± 0.83	2.13 ± 0.36	3.14 ± 1.65	2.98 ± 0.90	2.55 ± 0.55
**Adenosine**	0.02 ± 0.002	0.03 ± 0.01	0.02 ± 0.01	0.03 ± 0.01	0.02 ± 0.01	0.03 ± 0.01	0.02 ± 0.002
**Alanine**	1.93 ± 0.32	1.12 ± 0.41	1.07 ± 0.32	1.13 ± 0.26	1.58 ± 0.38	1.63 ± 0.63	1.32 ± 0.33
**Arginine**	8.13 ± 1.38	7.93 ± 1.13	7.05 ± 1.19	7.86 ± 0.99	9.24 ± 2.13	8.61 ± 1.38	8.68 ± 3.06
**Asparagine**	6.16 ± 1.95	6.29 ± 3.14	4.98 ± 1.60	4.50 ± 0.91	6.45 ± 1.49	6.36 ± 2.77	4.87 ± 1.71
**Aspartate**	4.05 ± 0.46	4.26 ± 0.89	4.82 ± 0.54	4.07 ± 0.70	4.99 ± 1.74	4.93 ± 1.13	4.73 ± 0.75
**Choline**	0.30 ± 0.06	0.27 ± 0.08	0.26 ± 0.07	0.32 ± 0.03	0.36 ± 0.08	0.36 ± 0.06	0.33 ± 0.11
**Citrate**	42.19 ± 3.66	45.40 ± 11.18	45.00 ± 7.84	38.56 ± 5.45	47.25 ± 14.11	46.30 ± 11.30	54.66 ± 4.86
**Formate**	0.44 ± 0.06	0.35 ± 0.05	0.19 ± 0.03	0.11 ± 0.03	0.12 ± 0.03	0.10 ± 0.02	0.33 ± 0.03
**Fructose**	182.30 ± 10.72	181.76 ± 44.98	178.11 ± 46.25	198.70 ± 20.86	223.95 ± 30.79	214.37 ± 17.61	245.75 ± 38.81
**Glucose**	184.22 ± 14.09	179.57 ± 50.39	181.88 ± 55.03	201.51 ± 30.41	237.43 ± 47.43	219.98 ± 14.00	285.86 ± 35.68
**Isoleucine**	0.09 ± 0.02	0.07 ± 0.02	0.08 ± 0.02	0.09 ± 0.05	0.10 ± 0.04	0.12 ± 0.04	0.09 ± 0.02
**Leucine**	0.09 ± 0.01	0.07 ± 0.02	0.08 ± 0.02	0.08 ± 0.04	0.09 ± 0.05	0.10 ± 0.03	0.09 ± 0.01
**Malate**	20.37 ± 2.50	24.88 ± 4.78	21.39 ± 5.24	21.62 ± 4.90	22.80 ± 4.15	23.96 ± 3.60	21.91 ± 2.77
**Malonate**	11.81 ± 3.56	10.16 ± 6.56	10.93 ± 2.58	10.94 ± 2.48	15.53 ± 4.95	15.84 ± 3.70	13.97 ± 3.58
**Phenylalanine**	0.32 ± 0.07	0.28 ± 0.07	0.31 ± 0.10	0.40 ± 0.45	0.27 ± 0.09	0.35 ± 0.07	0.39 ± 0.19
**Proline**	16.13 ± 8.06	12.01 ± 4.87	10.06 ± 2.68	13.95 ± 6.88	23.35 ± 12.09	19.18 ± 9.03	19.30 ± 8.70
**Succinate**	0.10 ± 0.01	0.14 ± 0.04	0.10 ± 0.04	0.11 ± 0.05	0.10 ± 0.05	0.15 ± 0.08	0.11 ± 0.03
**Sucrose**	190.35 ± 8.97	207.03 ± 37.58	196.17 ± 24.94	198.94 ± 15.44	210.75 ± 28.86	204.85 ± 5.57	166.27 ± 69.78
**Threonine**	0.29 ± 0.04	0.25 ± 0.06	0.24 ± 0.04	0.25 ± 0.07	0.30 ± 0.07	0.32 ± 0.07	0.29 ± 0.03
**Trigonelline**	0.10 ± 0.02	0.11 ± 0.02	0.09 ± 0.02	0.10 ± 0.02	0.14 ± 0.04	0.14 ± 0.03	0.13 ± 0.02
**Tyrosine**	0.08 ± 0.02	0.08 ± 0.03	0.08 ± 0.03	0.07 ± 0.03	0.10 ± 0.04	0.11 ± 0.04	0.10 ± 0.01
**Valine**	0.21 ± 0.04	0.17 ± 0.04	0.16 ± 0.05	0.22 ± 0.18	0.21 ± 0.08	0.24 ± 0.07	0.20 ± 0.04
**myo-Inositol**	15.87 ± 2.22	16.06 ± 5.20	15.24 ± 3.90	16.40 ± 2.16	17.64 ± 3.14	19.80 ± 2.41	21.37 ± 2.45

Table S1 - Juice metabolite concentrations in mM with +/- standard deviations for each time period

	Flavedo						
	W0	W1	W2	W3	W4	W5	W6
**4-Aminobutyrate**	1.12 ± 0.35	0.78 ± 0.21	0.62 ± 0.26	0.45 ± 0.12	0.50 ± 0.12	0.46 ± 0.25	0.40 ± 0.19
**Adenosine**	0.02 ± 0.01	0.02 ± 0.01	0.02 ± 0.01	0.02 ± 0.01	0.01 ± 0.01	0.02 ± 0.01	0.01 ± 0.01
**Alanine**	0.24 ± 0.05	0.26 ± 0.04	0.33 ± 0.07	0.23 ± 0.08	0.20 ± 0.05	0.21 ± 0.07	0.23 ± 0.08
**Arginine**	0.78 ± 0.21	0.88 ± 0.19	1.17 ± 0.23	1.01 ± 0.40	0.69 ± 0.38	1.12 ± 0.33	1.08 ± 0.45
**Asparagine**	0.54 ± 0.46	0.69 ± 0.69	0.58 ± 0.63	0.30 ± 0.38	0.18 ± 0.06	0.15 ± 0.13	0.11 ± 0.07
**Aspartate**	0.40 ± 0.20	0.57 ± 0.12	0.68 ± 0.25	0.50 ± 0.12	0.35 ± 0.13	0.34 ± 0.15	0.32 ± 0.13
**Choline**	0.26 ± 0.08	0.27 ± 0.05	0.34 ± 0.06	0.29 ± 0.10	0.24 ± 0.11	0.27 ± 0.12	0.29 ± 0.14
**Citrate**	0.31 ± 0.09	0.16 ± 0.07	0.20 ± 0.06	0.16 ± 0.07	0.32 ± 0.35	0.15 ± 0.06	0.22 ± 0.12
**Formate**	0.13 ± 0.03	0.09 ± 0.02	0.11 ± 0.03	0.07 ± 0.02	0.06 ± 0.04	0.07 ± 0.02	0.14 ± 0.04
**Fructose**	20.18 ± 3.48	22.35 ± 6.34	26.13 ± 4.45	28.26 ± 6.55	32.48 ± 9.45	35.46 ± 17.17	35.00 ± 12.48
**Glucose**	30.51 ± 11.66	31.90 ± 9.50	39.05 ± 8.60	44.20 ± 16.39	53.14 ± 16.75	48.49 ± 23.41	48.55 ± 22.33
**Isoleucine**	0.07 ± 0.02	0.06 ± 0.02	0.10 ± 0.03	0.08 ± 0.03	0.06 ± 0.03	0.08 ± 0.03	0.12 ± 0.06
**Leucine**	0.07 ± 0.02	0.07 ± 0.02	0.10 ± 0.03	0.08 ± 0.03	0.06 ± 0.03	0.08 ± 0.03	0.10 ± 0.05
**Malate**	2.61 ± 0.65	1.89 ± 0.41	2.01 ± 0.66	1.36 ± 0.44	1.07 ± 0.75	0.98 ± 0.42	1.59 ± 0.71
**Malonate**	1.34 ± 0.64	1.14 ± 0.14	1.53 ± 0.74	1.06 ± 0.54	1.03 ± 0.43	1.08 ± 0.44	0.98 ± 0.46
**Phenylalanine**	0.27 ± 0.05	0.32 ± 0.15	0.31 ± 0.06	0.40 ± 0.11	0.30 ± 0.10	0.32 ± 0.21	0.33 ± 0.10
**Proline**	5.10 ± 3.07	4.47 ± 0.89	6.12 ± 2.26	4.54 ± 2.28	5.25 ± 2.64	4.65 ± 2.77	5.76 ± 3.24
**Succinate**	0.05 ± 0.02	0.04 ± 0.01	0.05 ± 0.01	0.04 ± 0.01	0.03 ± 0.02	0.04 ± 0.02	0.06 ± 0.03
**Sucrose**	24.21 ± 12.34	26.07 ± 4.68	36.27 ± 8.98	22.34 ± 14.87	19.28 ± 8.94	13.34 ± 4.88	19.00 ± 12.01
**Threonine**	0.25 ± 0.06	0.26 ± 0.06	0.33 ± 0.05	0.27 ± 0.10	0.23 ± 0.06	0.27 ± 0.10	0.35 ± 0.14
**Trigonelline**	0.01 ± 0.01	0.01 ± 0.01	0.01 ± 0.01	0.01 ± 0.01	0.01 ± 0.01	0.01 ± 0.00	0.00 ± 0.00
**Tyrosine**	0.13 ± 0.01	0.13 ± 0.05	0.22 ± 0.14	0.15 ± 0.07	0.09 ± 0.05	0.12 ± 0.04	0.19 ± 0.12
**Valine**	0.08 ± 0.02	0.07 ± 0.01	0.10 ± 0.02	0.09 ± 0.04	0.07 ± 0.04	0.08 ± 0.03	0.12 ± 0.08
**myo-Inositol**	3.37 ± 0.52	3.87 ± 1.17	4.78 ± 0.37	4.46 ± 1.30	4.32 ± 1.28	4.29 ± 2.20	5.02 ± 1.76

Table S2 - Flavedo metabolite concentrations in mM with +/- standard deviations for each time period

	Albedo						
	W0	W1	W2	W3	W4	W5	W6
**4-Aminobutyrate**	0.78 ± 0.23	0.88 ± 0.37	0.44 ± 0.16	0.56 ± 0.17	0.60 ± 0.24	0.55 ± 0.23	0.49 ± 0.08
**Adenosine**	0.01 ± 0.01	0.01 ± 0.01	0.02 ± 0.01	0.02 ± 0.01	0.02 ± 0.01	0.02 ± 0.01	0.01 ± 0.01
**Alanine**	0.27 ± 0.09	0.34 ± 0.10	0.24 ± 0.09	0.26 ± 0.09	0.27 ± 0.05	0.25 ± 0.07	0.26 ± 0.04
**Arginine**	0.32 ± 0.09	0.46 ± 0.11	0.37 ± 0.12	0.53 ± 0.08	0.82 ± 0.21	0.57 ± 0.18	0.50 ± 0.15
**Asparagine**	0.31 ± 0.21	0.42 ± 0.48	0.17 ± 0.16	0.35 ± 0.52	0.21 ± 0.08	0.16 ± 0.09	0.11 ± 0.06
**Aspartate**	0.24 ± 0.11	0.43 ± 0.18	0.41 ± 0.22	0.43 ± 0.15	0.41 ± 0.12	0.27 ± 0.13	0.20 ± 0.09
**Choline**	0.17 ± 0.04	0.24 ± 0.02	0.17 ± 0.06	0.23 ± 0.04	0.37 ± 0.16	0.22 ± 0.07	0.21 ± 0.04
**Citrate**	0.26 ± 0.14	0.22 ± 0.11	0.25 ± 0.21	0.17 ± 0.08	0.29 ± 0.19	0.15 ± 0.05	0.19 ± 0.12
**Formate**	0.04 ± 0.01	0.03 ± 0.02	0.03 ± 0.01	0.04 ± 0.01	0.08 ± 0.05	0.03 ± 0.01	0.06 ± 0.01
**Fructose**	23.61 ± 3.46	26.54 ± 6.23	24.17 ± 5.86	39.03 ± 2.69	45.18 ± 8.16	40.83 ± 16.47	43.09 ± 6.81
**Glucose**	39.34 ± 5.42	45.65 ± 9.63	42.96 ± 9.53	67.74 ± 6.77	74.85 ± 20.81	67.44 ± 27.55	71.18 ± 16.55
**Isoleucine**	0.02 ± 0.01	0.04 ± 0.01	0.03 ± 0.01	0.05 ± 0.02	0.07 ± 0.03	0.05 ± 0.02	0.05 ± 0.02
**Leucine**	0.03 ± 0.01	0.05 ± 0.01	0.04 ± 0.01	0.05 ± 0.01	0.07 ± 0.01	0.05 ± 0.02	0.05 ± 0.01
**Malate**	0.76 ± 0.20	0.76 ± 0.55	0.76 ± 0.43	0.78 ± 0.33	0.97 ± 0.61	0.75 ± 0.30	0.70 ± 0.12
**Malonate**	0.97 ± 0.36	1.15 ± 0.24	0.90 ± 0.25	0.97 ± 0.28	1.15 ± 0.29	1.03 ± 0.35	1.03 ± 0.29
**Phenylalanine**	0.18 ± 0.07	0.31 ± 0.09	0.20 ± 0.11	0.42 ± 0.28	0.42 ± 0.13	0.24 ± 0.16	0.25 ± 0.06
**Proline**	3.29 ± 1.05	4.41 ± 1.18	3.44 ± 1.07	4.70 ± 1.33	6.31 ± 1.51	5.40 ± 2.69	5.37 ± 1.82
**Succinate**	0.01 ± 0.00	0.02 ± 0.00	0.02 ± 0.01	0.02 ± 0.00	0.02 ± 0.01	0.02 ± 0.01	0.02 ± 0.00
**Sucrose**	20.02 ± 8.18	26.68 ± 6.63	22.36 ± 7.45	16.47 ± 3.44	19.92 ± 5.36	11.83 ± 3.58	12.59 ± 3.19
**Threonine**	0.14 ± 0.04	0.33 ± 0.19	0.18 ± 0.03	0.20 ± 0.03	0.26 ± 0.03	0.22 ± 0.07	0.25 ± 0.04
**Trigonelline**	0.01 ± 0.01	0.01 ± 0.00	0.01 ± 0.00	0.01 ± 0.00	0.00 ± 0.00	0.00 ± 0.00	0.00 ± 0.00
**Tyrosine**	0.05 ± 0.02	0.07 ± 0.02	0.05 ± 0.01	0.06 ± 0.02	0.09 ± 0.05	0.05 ± 0.02	0.07 ± 0.01
**Valine**	0.03 ± 0.01	0.04 ± 0.01	0.03 ± 0.01	0.04 ± 0.01	0.07 ± 0.01	0.04 ± 0.02	0.05 ± 0.02
**myo-Inositol**	3.68 ± 0.51	5.15 ± 0.68	5.16 ± 1.29	6.90 ± 0.69	6.84 ± 1.53	6.40 ± 2.18	7.03 ± 1.53

Table S3 - Albedo metabolite concentrations in mM with +/- standard deviations for each time period

Brix and Acid % of Juice

	W0	W1	W2	W3	W4	W5	W6
**Brix**	10.29 ± 1.84	11.26 ± 1.70	11.23 ± 2.11	12.34 ± 1.99	13.41 ± 2.22	13.13 ± 1.09	12.96 ± 2.09
**Acid %**	0.77 ± 0.09	0.80 ± 0.22	0.79 ± 0.17	0.65 ± 0.14	0.73 ± 0.16	0.70 ± 0.10	0.70 ± 0.06
**Brix/Acid%**	13.68 ± 3.35	15.06 ± 4.76	14.70 ± 4.03	19.25 ± 2.56	18.68 ± 2.75	19.12 ± 2.70	18.52 ± 3.03

Table S4 - Brix and Acid% in Juice with +/- standard deviations for each time period
